# Physician Attributes That Matter Most: Results from a Qualitative Inquiry of Oncologists, Patients Receiving Oncological Care, and Medical Students

**DOI:** 10.3390/curroncol32060343

**Published:** 2025-06-11

**Authors:** Kimberly McMillan, Deborah Akurang, Paul Wheatley-Price

**Affiliations:** 1 Faculty of Health Sciences, School of Nursing, University of Ottawa, 451 Smyth Rd, Ottawa, ON K1H 8M5, Canada; 2 Department of Medicine, The Ottawa Hospital Research Institute, The Ottawa Hospital, University of Ottawa, Ottawa, ON K1H 8L6, Canadapwheatleyprice@toh.ca (P.W.-P.)

**Keywords:** physician attributes, relational inquiry

## Abstract

Background: Physician attributes significantly impact patient outcomes, satisfaction, and trust. Various attribute frameworks have been developed to help structure and guide undergraduate medical education and subsequent clinician practice; however, prioritization of these attributes vary by stakeholder (patients, physicians, medical students). Based on findings from two previous studies completed by the research team, we sought to understand the context in which individuals in these stakeholder groups prioritize particular physician attributes. We adopted a qualitative approach, conducting semi-structured interviews with patients (N = 11), doctors (N = 11), and medical students (N = 12), for a total sample of 34. Results: Thematic analysis of data resulted in the following five themes: caring, communicator, expert, professional, curiosity and open-mindedness. Central to our findings was the need for a positive, trusting provider–patient relationship, which was framed as the conduit to quality patient care (both receiving and providing). The attributes believed to support this central finding differed, noting that “caring”, “curiosity and open-mindedness” are not typical in physician attribute frameworks. Findings suggest there is a central guiding philosophy shaping what medical students, physicians and patients alike, need in the context of the provider–patient relationship, which transcends particular attributes. The guiding philosophy of relational inquiry is used to further situate study findings. Conclusions: Integrating a central guiding philosophy can add additional depth and nuance to attribute frameworks, ensuring considerations for qualities that transcend particular attribute characteristics, such as “caring” and “curiosity and open-mindedness” are also explicitly used to help structure and guide undergraduate medical education and subsequent clinician practice.

## 1. Introduction

Attributes of physicians greatly impact the quality of doctor–patient relationships, mediating the consultation process, treatment adherence, whether or not patients engage in health seeking behaviors, and ultimately impact patient satisfaction and even health outcomes [1,2]. The literature suggests that, broadly speaking, patients desire attributes of empathy, compassion, and caring from physicians [3,4]. Seminal works on the physician–caregiver relationship suggest that caregivers need to feel that they are a collaborator in care and associated decision making, yet also require empathy, support and care from physicians; in doing so, caregivers’ own health and wellbeing are better supported [5,6]. Physicians, nurses, and allied health professionals broadly value attributes of system leadership, lifelong learning, and interpersonal competencies [7].

In Canadian medical training, the Royal College of Physicians and Surgeons of Canada developed a framework of attributes that defines “abilities physicians require to effectively meet the healthcare needs of the people they serve” [2] (para1), abilities which are often referred to as physician attributes. There are seven attributes defined within this particular framework, referred to as the CanMED framework: medical expert, professional, communicator, collaborator, leader, health advocate, and scholar [2]. Medical expert sits at the center of the framework, upon which all other attributes bloom from.

Medical expert encompasses physicians’ abilities to actively engage in evidence-based knowledge, while mobilizing clinical skills which include, but are not limited to, the collection and interpretation of information, clinical decision making, and the execution of required diagnostic and therapeutic interventions, all performed in ways that uphold professional values [2]. Professional is understood as “clinical competence, a commitment to ongoing professional development, promotion of the public good, adherence to ethical standards, and values such as integrity, honesty, altruism, humility, respect for diversity, and transparency with respect to potential conflicts of interest” [2]. This particular attribute acknowledges the privilege physicians hold in society, and as such highlights the accountability required of physicians’ various intersecting levels. Communicator is understood as having the ability to engage in patient-centered therapeutics by communicating in ways that allow for the exploration of patient symptoms and utilizing active listening skills when exploring the potential for illness or disease [2]. Collaborator is demonstrated by sharing knowledge, perspectives, and responsibility, and demonstrating the will to learn alongside others and reach commonly held, patient-centered goals and outcomes. Collaborator requires that physicians understand the roles of other health professionals and navigate differences amongst interprofessional teams as they arise [2]. Collaborator extends beyond clinical care into education, scholarship, advocacy, and administration. Leader, according to CanMED, is defined as the ability of a physician to engage in “shared decision making for the operation and ongoing evolution of the healthcare system…locally, regionally, nationally and globally” [2]. The health advocate attribute underpins a duty to improve the health and wellness of patients, communities, and populations [2], while recognizing that health is not limited to the absence of disease, and includes aspects of health promotion, harm reduction, and social justice in the name of health equity. Lastly, scholar is understood as the ability to actively and continuously acquire knowledge across a physician’s career, serving as a role model for lifelong learning [2]. Scholar also includes the ability to develop expertise through the best available evidence and contribute to advances in scholarship in a physician’s field of expertise. The CanMED framework guides undergraduate physician training within Canadian medical schools and guides ongoing professional development for practicing Canadian physicians. But are these attributes also valued by key stakeholders in the Canadian healthcare system: patients, caregivers, nurses, allied health, physicians, and medical students? This particular question guided a series of three research studies. The findings from the first two studies are briefly described below, with the foci of this paper reporting on findings from the third, and final study in this series.

### Foundational Research: Exploring the CanMED Framework

The first of a series of three planned studies, which set out to examine the CanMED framework in the context of healthcare stakeholders’ own beliefs about physician attributes, was completed in 2018 and published in 2020 [8]. Stakeholders, including medical students, doctors, nurses, patients, and caregivers from a single academic teaching hospital and affiliated medical school, were quantitatively surveyed to answer the following question: ‘using one word only, what do you think is the single most important attribute a physician should have’. Answers were mapped onto the CanMED’s attribute framework. The most common answers across all stakeholder groups reflected a ‘caring’ domain with 58% of 362 responses falling into this domain. Examples of attributes pooled into a caring domain included ‘compassion’ and ‘empathy’. Though integrated throughout the CanMED’s framework, there is not a specific attribute designated to caring, despite caring being central to the needs of patients and caregivers requiring physician care.

The second study, completed in 2019 and published in 2023 [9], hypothesized that respondents in study one may have been assuming other, underlying qualities in physicians when they prioritized “compassion” and “empathy”. To test this, we asked respondents to rank the eight important physician attributes found in study one. In total, 375 respondents participated in the survey. “Knowledge” and “competence” (which mapped to the CanMED attribute, medical expert) were the most popular answers among all groups except medical students. Study one identified compassion as a highly valued attribute; however, findings from study two suggest that this may be with the assumption that a physician is knowledgeable and competent. Interestingly, when examining the subgroup data, only the medical students were outliers in still selecting the caring domain answers as the most important. All other groups (patients, caregivers, nurses, doctors, and allied health professionals) ranked competence or knowledge as the top answer most commonly.

In this third and final study of the series, we adopted a qualitative approach with the use of semi-structured interviews with patients, doctors, and medical students to understand the contexts in which individuals in these stakeholder groups prioritize physician attributes. In asking participants what they believed to be the most important attribute of a physician, this study then used a semi-structured interview to understand reasons and both personal and professional experiences that led to those choices. This study is reported in accordance with the Consolidated Criteria for Reporting Qualitative Research (COREQ) checklist, which is provided as a Supplemental File S1.

## 2. Methods

Methodology was qualitative [Interpretive Description] [10], with semi-structured interviews being utilized to answer the research questions by way of interpretive analysis [10]. This particular methodology allows for an in-depth understanding of experiences, perceptions, and beliefs, eliciting rich description [11], which is important when understanding the complexity of how people come to value particular attributes of health professionals. Semi-structured interviews allow researchers to explore complex experiences but also provide focus that guides the interview to ensure research questions are answered in the data collection process [12].

Participants were from three of the targeted stakeholder groups explored in the previous two studies conducted by the research team [8,9]: 1st or 2nd year medical students, physicians and patients. First and second year medical students were chosen (as opposed to year 3 and 4) because they have less formal socialization into medicine, which helps further distinguish their stakeholder perspective from that of practicing physicians who participated. Those who participated met the following inclusion criteria: training in Ontario (medical students); providing care in Ontario (physicians); receiving care in Ontario (patients); able to participate in an interview in English; and have access to a working telephone and/or internet connection as all interviews were conducted virtually via Microsoft Teams. Exclusion criteria were as follows: training outside of Ontario(medical students); providing care outside of Ontario (physicians); receiving care outside of Ontario (patients); unable to participate in an interview in English; and no access to a working telephone and/or internet connection. Recruitment occurred through clinical, personal, and academic networks in a large urban city in Ontario. Initial recruitment occurred through a large teaching hospital in Ontario and an affiliated medical school, then the research team’s personal, academic, and clinical networks were utilized. Recruitment material consisted of three recruitment posters, one for each stakeholder group. Recruitment methodology used both purposive sampling [13,14] and snowball sampling [10]. Purposive sampling was used to recruit medical students, patients, and physicians that could answer the research question(s). Medical students were provided recruitment information in lectures; however, it was made explicit that their participation was voluntary, and in no way tied to any course or associated grade. Recruitment posters were posted in various locations at the large teaching hospital. Patients were reminded of the invitation to participate after their scheduled appointments and were provided with a recruitment poster if they chose so. Recruitment was further supported via snowball sampling techniques—recruited patients, medical students, and physicians were invited to share recruitment information with others that they believe could help answer the research questions.

Data collection occurred between June and August of 2021. All participants received a written consent form prior to participation, which was reviewed and discussed at the beginning of each interview. At that time, verbal consent was received. Data was collected by a research student [DA] under the supervision and training of the researcher leading the qualitative component of this study [KM]. KM worked with DA to develop understandings of qualitative research tenants and process, practiced interviewing techniques with DA prior to data collection, and debriefed with DA at various points during data collection. Participants were asked the following questions: If you could only use one word to describe the most important attribute in a doctor, what would it be?; Can you tell me about why you believe this is the most important attribute?; and If there a specific encounter with a doctor or doctors that has led you to believe this is the most important attribute(s), could you tell me more about this? Interviews ranged in length from 20 to 45 min. The research team believed the research questions have been answered richly (as per chosen methodology) after the completion of 34 interviews, when data collection stopped. In the chosen methodology, Interpretive Description [10], richness in answering the research questions is achieved when researchers move beyond simply identifying themes and patterns to developing new insights and discoveries, which the authors believe has been performed by situating the data beyond existing attribute frameworks and providing new insights from the study data in the Discussion Section. Interpretive Description methodology also emphasizes the importance of considering data that do not fit the dominant themes or patterns [10], which is demonstrated by the development of additional thematic findings that extend beyond the utilized CanMED framework.

Interview audio files were analyzed by DA and KM directly with no verbatim transcription. This method of analysis provides a much more immersive analytical process with an opportunity to interpret emotions and tone. Using an inductive approach, recordings were listened to multiple times by the study team. Reflections on the data, related to the aims of the research, were captured and notes of salient points and preliminary insights were made. In-depth analysis of each participant’s interview was then performed. Subsequent coding was both inductive and deductive, the coding framework is provided as a Supplemental File S2. Inductive coding meant that specific segments of discourse providing partial answers to the research question were analyzed and interpreted, for example, contextualizing individual’s rationales for their chosen attribute. Deductive analysis mapped chosen attributes to the CanMED domains, where such domains existed, noting that participants’ responses that did not align with CanMED domains were also coded and are discussed in both the Results and Discussion sections of this paper. Interpretive syntheses of each participant’s interview were written, then read together to achieve an analysis across participant narratives, firstly per stakeholder group (patients, medical students, and physicians), then across each stakeholder group. When differences in opinions of data meanings emerged between analysts [DK & KM], discussion ensued until a consensus was reached.

The study received local ethics board approval. All data and project documents were digital in nature, encrypted, and password protected. Participant’s identity was known only to DA and KM. It was up to participants whether they chose to disclose to others that they participated in this study.

## 3. Results

A total of 34 people participated: 12 medical students, 11 physicians, and 11 patients. Medical students were in year 1 or 2 of an accredited medical school located in a large urban city in Ontario. Physicians were medical oncologists, who practice in Ontario in both inpatient and outpatient oncology care areas. Patients were those receiving oncological care at a cancer center in Ontario, with varying cancer diagnoses and prognoses. Thematic analysis of data resulted in the following five themes: (1) caring; (2) communicator; (3) expert; (4) professional; and (5) curiosity and open-mindedness. Three of the themes (communicator, expert, and professional) can be explicitly mapped to particular attributes within the CanMED framework mobilized in this study, whilst two themes (caring and curiosity and open-mindedness) cannot. This will be explored in the Discussion Section of this paper. After immersive data analysis, it was determined that disaggregating the data by way of each stakeholder group (medical students, physicians, and patients) was not necessary, nor would doing so enhance our understanding of the findings. Tensions, incongruence, or inconsistencies between stakeholder group findings did not arise as they related to the central thematic findings, which in and of itself may be considered a finding. There was uniformity in the ways all stakeholder groups understood the significance of attributes in relation to provider–patient relationships, provider–caregiver [patient’s loved ones] relationships, and patient care delivery. We have been deliberate in data presentation, ensuring representation from stakeholder groups reflective of each thematic finding are all represented. We also offer relative perspectives and key findings from each significant stakeholder group.

### 3.1. Theme 1: Caring

Caring was the most commonly described attribute among all participants. However, medical students and patients represented the majority of respondents aligning with the caring domain, physicians represented the minority in the selection of this particular attribute. As defined in our two previous studies, the caring domain describes a physician’s role as an empathetic and emotional supporter of patients and their loved ones. The majority of participants reported caring as the most important attribute, believing that, although competence and medical expertise are baseline qualifiers of a physician, attributes like “compassion” and “empathy” are required to develop an excellent doctor–patient relationship, a cornerstone of healthcare provision. For example, one participant said:

“I think it [caring] contributes very heavily to the creation of the strong patient-physician relationship which I feel like should be the foundation of the healthcare transaction”.

(medical student)

Participants provided various responses when discussing the experiences that led to them choosing caring as the most important attribute. Medical students and physicians discussed the limitations of medicine and available treatments. One physician, reflecting on a patient who had rapidly deteriorated, commented that although they could not cure the patient, demonstrating compassion made the patient feel listened to and cared for. One medical student offered the following:

“a lot of the time in medicine there is not always a lot that you can do [medically; curatively], but being compassionate to somebody’s situation is always possible”.

(medical student)

Medical students and physicians alike selected attributes in the caring domain because they recognize the value of caring, particularly when they feel their medical expertise can only go so far, especially in cases of terminal conditions with limited or already exhausted treatment options.

Patients who chose caring as the most important attribute expressed the need to be recognized as more than a medical problem. Being a patient was described as a challenging experience that can foster a constellation of negative emotions. Patients in this study described how provider–patient interactions shape one’s outlook of their health journey. When effort is made to care for the person and not only the condition, patients reported feeling valued and seen:

“I think for some patients, a lot of patients actually…being seen as an individual, even if you’re going through something thousands of other people have gone through with this doctor is extremely important. Because, you feel that you’re then valued … I think that comes through empathy”.

(patient)

Physician attributes within a caring domain provided patients with a sense of personhood extending beyond their clinical diagnosis, while attributes within a caring domain were understood by medical students and doctors as means to create strong provider–patient relationships, notably in the context of patient disease trajectories where curative interventions are no longer available.

### 3.2. Theme 2: Communicator

The attribute of communicator centers on the physician’s ability to provide and receive information. It was the second most common theme overall, with patients representing the resounding majority of these responses. Participants who chose communicator as the most important attribute of a physician believe communication improves the provider–patient relationship, which in turn benefits the quality of patient care received. All participants who chose this as their top attribute recognized that the relationship between a physician and a patient can sour without proper communication. One participant reported that a lack of communication from their physician and between healthcare professionals following their surgery caused preventable and unnecessary pain:

“That [poor communication] was most detrimental to my health. I couldn’t plan. I had no way to anticipate how bad it [post-operative pain] was going to be or how I could manage it”.

(patient)

Participants provided different rationales for communication as their chosen most important physician attribute. Generally, those with a medical background shared common values in their narratives, while comparatively, patients offered slightly different shared values in their stories. The few medical students and physicians who chose communicator as the most important attribute did so because they had witnessed how advantageous communication was to successful medical team functioning and patient encounters. One physician participant recalled witnessing a particular communication style, the use of an analogy, while shadowing a senior physician who used analogy when explaining the rationale for stopping chemotherapy to patients. The physician recalled how “that seemed to alleviate a lot of stress on the patient’s part”. This example demonstrates how utilizing different styles of communication can enhance care delivery.

Participants in the patient group who chose this as the most important attribute expressed the desire to be heard. Almost all patients acknowledged their primary concern was not feeling “rushed” or being “interrupted” during provider–patient interactions. One participant described an encounter with a physician who interrupted them by putting words in their mouth. Although the physician was correct on occasion when interrupting, she was frustrated because the physician’s poor communication style left her confused and made her feel like she had no input into making decisions regarding her healthcare. Other patient participants reported similar experiences of poor communication, whereby participants reported coming out of a physician appointment having learned nothing and feeling disengaged when communication had become unidirectional. One participant shared this process of shutting down:

“She [physician] wasn’t listening to what I said and so automatically I shut down, I just didn’t want to talk to her anymore”.

(patient)

Patient participants who chose communication as the most important physician attribute shared the desire for physicians to recognize and address their unique needs, believing this is accomplished only when physicians enact strong communication skills, and make conscious efforts to listen. Medical students and physicians who chose communication valued how communication can foster enhanced patient outcomes, both physically and emotionally.

### 3.3. Theme 3: Expert

Expert refers to the role of the physician as a provider of current and relevant medical care. Of the five themes identified, this ranked third overall. Respondents who chose this attribute were overwhelmingly doctors and medical students, with only one patient providing expertise as their most valued physician attribute. Participants expressed similar rationales for choosing expertise, highlighting that expertise is what they believe fosters trust in the provider–patient relationship. Doctors and the medical students expressed their desire to honor and uphold the trust a patient has in a physician to manage their health [and illness] properly and in good faith, which according to these participants, can only be done through the attribute of expertise. Physician and medical student participants described expertise as the all-encompassing attribute necessary to support patients in reaching their health goals. While doctor and medical student participants recognized the value of other attributes, they described them as secondary to that of experts:

“If medical expertise is not there, then you’re not a doctor”.

(medical student)

One physician participant reported witnessing doctors who had good patient relationships (fostered through the attribute of caring) in which patients were happy with their care; however, in some of these circumstances the participant witnessed potential deficits in treatment management due to a lack of expert knowledge on behalf of the provider. For this reason, this participant emphasized that, while a patient’s comfort is important (feeling cared for), providing quality medical care derived from a physicians’ expertise should be the center of physician’s work. Another physician participant reiterates this hierarchy, suggesting that so long as expertise is demonstrated, other [caring] attributes may not even be necessary [in their perspective]:

“I’ve had interactions with doctors who have not been empathetic, but in my mind, as long as they are treating somebody properly and thinking of the right things [via their level of expertise], and not missing things that are dangerous. [That is what] I would say [is] the most important.”

(physician)

The sole patient participant who chose this attribute as most important reported that expertise manifested in physician confidence, and that confidence was necessary for the patient to trust the physician when making treatment decisions. For instance, following the patient’s cancer diagnosis, they stated how they needed their doctor to be confident in treatment decisions:

“What I needed is a doctor who is going to help me treat the cancer, [who] was confident and stable. … confident that you know we can do this, we can do that. This is what I mean by being confident … knowing what to do [medically]”.

(patient)

The participant further explained that the trust created through a doctor’s confidence [which in the participant’s mind demonstrated expertise] reduced the patient’s feelings of anxiety and stress.

### 3.4. Theme 4: Professional

Professional was the fourth most selected attribute and is defined as upholding professional codes of conduct and standards of practice, which include a physician’s ability to function within a team. Patient participants did not choose this attribute. Physicians represented the majority of those who chose this attribute, while only one medical student chose this particular attribute. This group of participants reported professional as the most important attribute because they believe this attribute cultivates the trust-based relationship between patients and physicians necessary to deliver quality care.

Physician participants discussed their responsibilities to understand patient goals and their duty to uphold the trust individuals instill to care for them, and believed these actions were most in line with the attribute of professionalism. Participants report not having one particular experience from which they founded their decision, instead, witnessing and having multiple personal encounters with patients has demonstrated how enacting professionalism displays respect for patients and helps develop provider–patient relationships. One participant shared that professionalism in medicine is exemplified when patients feel they can rely on their physician without fearing abuse of power, negligence, or disrespect. To them, physicians are held to a standard of excellence, and acts of professionalism are how they uphold that standard:

“It’s a relationship where there is assurance that the patient’s goals, priorities, wishes are going to be … what’s driving the agenda, what’s driving the relationship, what’s driving the treatment plan and then … there’s competence around being able to deliver on what’s being discussed or what’s being agreed upon”.

(physician)

The participant in the medical student group who chose this attribute as the most important reported that their experiences shadowing physicians led them to choose this attribute. They expressed an admiration for doctors who were respectful towards their patients, this participant associated respectfulness with professionalism. The participant recalled witnessing a family doctor rushing to meet with a patient after they told the doctor that the patient was in a hurry to pick up their child. The participant noted the doctor’s respectful response to the medical student when relaying this information, and the priority the physician gave this patient in light of their caregiving responsibilities demonstrated high levels of professionalism.

The attribute of professional, as understood by these participants was deemed significant through repeated witnessing of acts that positively impacted patient care, notably through deliberate acts of trust building within the provider–patient relationship.

### 3.5. Theme 5: Curiosity and Open-Mindedness

The final theme, which we’ve entitled curiosity and open-mindedness, was reported only by doctors and medical students, of which medical students were the primary respondents. “Curiosity”, “open-minded” or “non-judgmental” were the predominant responses for this category. According to these participants, when a physician is curious, they seek knowledge, which medical students believe positively impacts patient care, and outcomes. Medical student participants reported choosing this attribute because they witnessed physicians who used curiosity to navigate patient encounters and saw how beneficial it was to clinical care. One participant observed a family physician demonstrating curiosity and shared how the doctor openly admitted to the patient that they were unsure what was happening to them. Rather than sending the patient to another physician, the physician made a follow-up appointment, subsequently taking the time to do the research and come back for the next appointment with the necessary knowledge to explain to the patient what they believed was going on. These participants chose curiosity as the most important attribute because they believed being a doctor is a job that requires lifelong learning, and they believe curiosity is the attribute that drives physicians to seek knowledge and find answers that serve their patients, for the duration of their medical careers.

Open-minded and non-judgmental were the attributes chosen by physician participants. The participants defined this attribute as the attitude or composure a physician embodies when interacting with patients and colleagues. They expressed that being open-minded means not viewing patients through a rigid lens of risk factors and not defaulting to a preconceived understanding or bias. One participant believed that open-mindedness is the most important attribute because she had witnessed how being close-minded damaged provider–patient relationships and provider–provider relationships. For instance, the participant worked with a colleague who was rigid about the weight and body mass index [BMI] of their patients, and when a patient asked why they got uterine cancer, the physician told the patient it was because they were obese. Unfortunately, they witnessed the same physician demonstrate this attitude towards multiple patients, which was detrimental to the provider–patient relationship, as noted in the following quote:

“A few patients refused to even speak with [the doctor], would only talk to the resident. …which is not a very good patient provider relationship”.

(physician)

Once again, as seen in previous themes, physicians and medical students chose their particular attribute because they value its role in developing positive, trusting provider–patient relationships, which they associate with quality patient care delivery.

## 4. Discussion

The results provide insights into how a sample of physicians, medical students and patients come to value a certain physician attribute over others. This study adds to the existing understanding of physician attributes, especially through suggesting that regardless of the attribute discussed by participants, the value associated with attributes amount to desiring the same relational outcome: medical students, physicians, and patients alike need positive, trusting provider–patient relationships, which was framed as the conduit to quality patient care transcending particular attributes. These findings suggest a more philosophical orientation to understanding quality patient care, providing an opportunity to further examine higher level philosophical orientations of quality patient care in the contexts of undergraduate physician training, and ongoing professional development focused heavily on attribute acquisition.

Albeit not surprisingly, the majority of participants told us that caring was the most important attribute. The sample of participants that prioritized this attribute included physicians, medical students, and patients, in a relatively balanced ratio. This particular finding is well established in and supported by the literature. Firstly, this is consistent with the first publication in our series of studies [8]. Additionally, Dopelt and colleagues [15] found that patients and physicians alike, when asked to define attributes of a “good doctor”, described “humanness” (p. 73). A recent systematic review [16] highlights the significance of empathy as a physician attribute of caring; however, notes that across the literature reviewed, empathy declines over the trajectory of medical school. Our data reflects findings from medical students in years 1 and 2—wherein empathy remains relatively high [16].

When mapping caring as an attribute to the CanMED framework [2]—caring, in and of itself is not a standalone competency. The seven competencies include: medical expert, communicator, collaborator, leader, health advocate, scholar, and professional. Other global frameworks also lack caring as a standalone core competency. For example, the United States Medical Licensing examination [17] has the following seven core competencies: medical knowledge/scientific concepts; patient care: diagnosis; patient care: management; communication; professionalism; including legal and ethical issues; systems-based practice; including patient safety; and practice-based learning. The United States Accreditation Council for Graduate Medical Education [18] describes six fundamental competencies which also do not have caring as a standalone competency. Historically, caring has been situated as an implicit attribute of those who choose to work in health professions [19]. Implicit attributes in health professions, such as caring, are often subject to something described as “hidden curriculum” [20]—values and norms that are not explicitly articulated in core curriculum and associated with learning outcomes but rather assumed to be role modeled throughout a student’s educational experience. However, caring as a taken-for-granted attribute in the health professions is problematic, because it purports the assumption that all practitioners know how to demonstrate care and engage in caring acts in their daily practice; however, for many, this is a skill that must be taught, practiced and role modeled [21]. The findings from this particular theme provide an opportunity to reflect on the merit of incorporating care as an explicit attribute for physician practice.

The second most selected attribute, communication, was selected disproportionately by patients in comparison to physicians and medical students. The literature suggests that communication from physicians is of paramount importance to patients and impacts their health experiences. Patients describe needing clear, effective, and robust communication from physicians, along the entirety of the wellness–illness continuum [22]. Poor physician communication results in feelings of stress, anxiety, uncertainty, and for some patients, even psychological distress [22]. In contrast, strong physician communication results in positive patient outcomes ranging from therapy adherence to enhanced self-management skills [23]. What is interesting to note in our data is only 1 (of 11) physician’s described communication as the most important attribute, while four times as many patients did. This was the biggest discrepancy range between responses from stakeholder groups in the coded data. One reason to explain this discrepancy may lie in the current conditions of the healthcare system, notably in Canada where the study has taken place. There is currently a health human resource crisis in Canadian healthcare [24], meaning vacant positions are at all-time highs [25], resulting in incredibly high workloads for those currently working within the system [26]. Communication requires time, time that some healthcare providers, including physicians, do not feel they currently have enough of to perform communication well without sacrificing other aspects of care delivery [27]. Perhaps these circumstances have potentially downgraded the importance of this attribute for physician participants who possibly turned their attention to attributes they perceive to have more control over, like caring or expert.

The third most selected attribute of our study, expert, provided both expected and unexpected findings. Only one patient deemed expert to be the most important attribute of a physician, while most of the participants that chose this attribute were physicians themselves. Typically, it has been asserted that patients derive trust in the provider–patient relationship through a provider’s level of expertise and competence [28] but do ultimately value strong physician communication skills that allow patients feel listened to [29]. Our second study [9] had identified that medical knowledge and expertise was ranked higher than caring or communication, when participants were asked to rank these attributes. A unique finding was that only 1 (of 12) medical student chose this attribute as the most important. This begs the question—what happens between medical school and independent practice to cause this shift in attribute prioritizing? Socialization in health professions training readily impacts learners’ values, beliefs and priorities [30], perhaps socialization during medical training results in the re-prioritizing of these attributes, whereby expert may be an attribute readily visible and role modeled in student learning environments. On the contrary, as noted in some of our findings, medical students might encounter an event in which they question if expertise was exemplified, which may challenge learners to reflect on the significance of expertise in medicine, potentially increasing the value they place on this particular attribute.

Professional was the fourth most selected attribute—patients did not choose this attribute, only physicians and one medical student did. This finding is not surprising, as professionalism is a cornerstone of professional licensure for physicians and is intrinsically linked with upholding patient safety [31] and is a foundational component of medicines ongoing social contract with society [32]. The literature exploring patients’ understandings of professionalism highlight that patients often frame physician professionalism as synonymous with caring [33], which could explain why patient participants in this study did not choose professionalism but rather opted for caring as the most important attribute (as noted in theme one).

Our fifth theme, curiosity and open-mindedness, emerged as a standalone attribute not explicitly attached to any of the CanMED [2] domains used to theoretically frame this study. This particular attribute could potentially be implicitly linked to the expert attribute, as often curiosity drives clinicians to strive for expertise in their practice, but we believe it is important to distinguish curiosity and open mindedness as a stand-alone attribute that can be mobilized in concert with all desirable physician attributes.

Overarching all five themes and spanning responses from all participant groups (medical students, physicians, and patients), was the need for a positive, trusting provider–patient relationship, which was framed as the conduit to quality patient care (both receiving and providing); however, all that differed in responses was what particular attribute participants believed would be the means to that end. What this may suggest, is that at a higher level of abstraction [higher than attributes], there is a central guiding philosophy shaping what medical students, physicians, and patients alike, strive for giving and receiving in the context of the provider–patient relationship. We believe that guiding philosophy may be relational inquiry [34].

Relationality in healthcare delivery is most often understood within the sole context of the provider–patient relationship—relational inquiry is not this. As a guiding philosophy, relational inquiry is understood as a deliberate, conscious and ongoing focus of attention on “what is going on at and between the intrapersonal, interpersonal, and contextual levels of healthcare situations” [34], with the understanding that these various interplays shape all individual’s experiences and actions in healthcare situations, often in unique ways.

Relational inquiry is not prescriptive, it does not describe in detail what actions are, and are not deemed as relational in nature, rather it serves as a philosophy guiding all [inter] actions within healthcare. Relational inquiry scholars ask health practitioners and those who educate them, “what if we understand relation as the most fundamental, yet simultaneously advanced aspect [of practice]” [35], and strive to make that understanding explicit, as opposed to being implicit and assumed, often hidden within curriculum instead of being the central guiding principle of curriculum. We will use one of our findings to exemplify this philosophy in practice.

In our fifth thematic finding, curiosity and open mindedness, we described a physician participant witnessing how being close-minded and lacking curiosity damaged provider–patient relationships. Furthermore, the physician also recalled how physician judgements, particularly as they pertain to patient body weight and disease causality were made explicitly known to a patient and as such, were detrimental to provider–patient relationships. The physician participant recalls witnessing the same colleague demonstrate this attitude towards multiple patients.

From a strictly attribute-focused examination, the reader can deduce that the physician colleague described in the vignette lacked this particular attribute, reflecting a deficit-based approach in our understanding. This understanding is derived from an interpersonal lens only. When reframed from a relational inquiry standpoint we can deconstruct the vignette by exploring what may be happening at intrapersonal, interpersonal, and contextual levels.

When exploring what may be happening at an intrapersonal level of relation, relational inquiry would ask that we examine what may be going on within the physician. The literature notes that physicians experience a plethora of psychological harm [36,37], which at times, impacts their engagement in the provider–patient relationship. When examining what may be happening at the interpersonal level of relation, the literature notes that eight out of ten Canadian physicians report experiencing intimidation, bullying, harassment, and microaggressions within their place of work [36], which can also negatively impact the provider–patient relationship [38]. When exploring contextual levels of relation, we can inquire into the workplace culture [also referred to as organizational climate], and how it may contribute to ignoring or accepting these types of provider–patient interactions. It is repeatedly noted that overcrowding, underfunding, and health human resource crises create circumstances in which the patient–provider relationship is at risk [39]. Furthermore, mobilizing relational inquiry when examining physician attributes in practice allows us to examine how contextual level issues may be contributing to what is happening at the intrapersonal and interpersonal levels. Relational inquiry acknowledges that all three are interconnected and must be explored collectively to fully understand people’s experiences within healthcare.

This re-analysis is not extensive, providing only a few examples to stimulate thought and discussion. The purpose of this re-analysis using relational inquiry is not to rationalize or condone, but rather to gain a fulsome understanding of why desirable physician attributes may not be embodied, and in doing so, provides opportunity to reflect on what strength-based approaches may be taken to support practitioners in complex care environments that embody these important attributes. Relational inquiry situates attributes within a complex interplay of intrapersonal, interpersonal and contextual levels, which provide opportunities to explore how such attributes can be fostered at each of these intersecting levels.

Relational inquiry as a guiding philosophy also invites curiosity and open-mindedness, the fifth theme within our findings. Some health scholars have suggested that mobilizing relational inquiry as a guiding philosophy allows for the cultivation of relational consciousness, particularly as it pertains to social justice [35]. Although social justice was not explicitly identified within our data, there are urgent calls to more comprehensively embed core principles of social justice into all health profession’s education [40], with some calls coming directly from medical students themselves [41]. Examining attributes embedded within medical education and practice frameworks through a lens of relational inquiry may provide a robust opportunity to integrate social justice knowledge and eventual praxis into the embodiment of all desirable physician attributes.

The purpose of this paper is not to suggest attribute frameworks do not serve an important role in medical education and practice, rather that the purpose was to explore potential ways of re-imagining the positionality of attributes in how we understand medical education and practice. Our data suggests that all desirable physician attributes support one common goal, one that is grounded in relational inquiry. There is a place for tangible, measurable structures to guide medical education and practice, such as the CanMED framework [2]. There is also a place for higher level guiding philosophy, and that relationship has the capacity to be synergistic. Utilizing relational practice as a guiding philosophy in medical education and practice can serve to make caring more explicit, and may be utilized as means to maintain, or even increase, medical student empathy, which, as noted earlier, declines during medical school [16]. Relational inquiry philosophy may also further guide how particular attributes that frame medical education and practice can be understood and actualized in clinical practice, in ways that uphold the central finding of the research presented in this paper: Physician attributes serve as means to obtain positive, trusting provider–patient relationships, with the central goal of delivering (physicians and medical students) and receiving (patients) quality patient care. We believe these findings have important implications for medical education and practice. Our findings support the revisiting and potential reimagining of attributes in physician training and professional development. They also provide an invitation to examine how highly relational philosophies may also contribute significantly to learner and professional needs. Future research could further explore how fundamental principles of relational inquiry already exist in physician training and professional development, as well as ways that such principles could be increasingly integrated or made more explicit.

### Limitations

This study is not without limitations. The sample for each category of participant (physician, medical student, and patient) is homogeneous. Patients accessing a variety of clinical services [outside of oncology] may offer more diversified perspectives and provide insights into different areas of medicine. Geographically, the data set was confined to one Canadian province, offering only a small window into a vast country. Future research could expand upon the findings presented in this paper by examining physician attributes at a national level, and by expanding data collection methods to include nurses, allied health professionals, family/caregivers, medical students in later years of study, and residents and fellows working in a variety of clinical environments and specialties. There was also a delay in publication of results, resulting from competing research demands of the first author. Analysis was ongoing and returned to throughout the time of collection to publication, as supported by the methodology [10].

## 5. Conclusions

The study findings and discussion presented in this paper examine what a sample of Canadian physicians, medical students, and patients deem to be the most important attribute in a physician and how participants came to value one particular attribute over others. The CanMED framework [2] helped the research team structure and code findings, while the data also provided opportunities to look beyond attribute frameworks to explore higher-level philosophies that can guide the embodiment of physician attributes. Central to our findings was the need for a positive, trusting provider–patient relationship, which was framed as the conduit to quality patient care, regardless of what particular attribute participants spoke about. Study findings suggest the presence of a more philosophical orientation to quality patient care, which can be further understood by examining the role of attributes in fostering that care through a highly relational guiding philosophy.

## Data Availability

The data presented in this study are available on request, as aggregated, de-identified data sets from the corresponding author due to research ethics involving human subjects.

## Supplementary Materials

The following supporting information can be downloaded at: https://www.mdpi.com/article/10.3390/curroncol32060343/s1, S1: COREQ checklist; S2: Coding framework. (See Ref. [42]).
