# Supplementary material for: Physician Attributes That Matter Most: Results from a Qualitative Inquiry of Oncologists, Patients Receiving Oncological Care, and Medical Students
Source: Curr Oncol. 2025 Jun 11;32(6):343. doi: 10.3390/curroncol32060343 (PMC12191522; doi:10.3390/curroncol32060343)
Supplement: Supplementary file 1 [file curroncol-32-00343-s001.zip › curroncol-3591016-supplementary.pdf]

S1: COREQ

Domain 1: Research team and reflexivity

Personal Characteristics

| | |
|---|---|
| 1. Interviewer/facilitator. Which author/s conducted the interview or focus group? | D.A |
| 2. Credentials. What were the researcher's credentials? E.g. PhD, MD | Undergraduate student and Research Assistant, who received qualitative data collection training from K.M., and regular debriefs following participant interviews. |
| 3. Occupation. What was their occupation at the time of the study? | Research Assistant |
| 4. Gender. Was the researcher male or female? | Although we acknowledge the original COREQ terms are not inclusive of all genders, one of our researchers identifies as male (P.W.P), and two identify as female. (D.A, K.M) |
| 5. Experience and training. What experience or training did the researcher have? Relationship with participants | D.A received training on qualitative research from K.M, an established qualitative researcher. Training included the history of qualitative research, central guiding philosophies, role in knowledge production as well as pragmatic considerations for conducting qualitative interviews and analysis. This included how to engage with participants when discussing sensitive or emotive topics and how to be reflexive of personal bias and assumptions. |
| 6. Relationship established. Was a relationship established prior to study commencement? | D.A was the contact for recruitment, therefore established initial researcher-participant relationship through email exchange and garnering of consent. |
| 7. Participant knowledge of the interviewer. What did the participants know about the researcher? e.g. personal goals, reasons for doing the research | Participants knew the Research Assistant was an undergraduate student who was working closely with K.M and P.W.P. Participants were also made aware of the Research Assistant's wishes to pursue medical school and was very interested in the study topic. |
| 8. Interviewer characteristics. What characteristics were reported about the interviewer/facilitator? e.g. Bias, assumptions, reasons and interests in the research topic | The reasons and interests in the research topic; bias and assumptions were discussed on an ongoing basis with K.M through debriefs and reflexive note taking. |

Domain 2: study design

| | |
|---|---|
| 9. What methodological orientation was stated to underpin the study? e.g. grounded theory, discourse analysis, ethnography, phenomenology, content analysis | Sally Thorne's Interpretive Description. |
| 10. Sampling. How were participants selected? e.g. purposive, convenience, consecutive, snowball | Purposive and snowball sampling occurred through clinical, personal and academic networks. |
| 11. Method of approach. How were participants approached? e.g. face-to-face, telephone, mail, email | Medical students were provided recruitment information in lectures; however, it was made explicit that their participation was voluntary, and in no way tied to any course or associated grade. Recruitment posters were posted in various locations at the large teaching hospital. Patients were reminded of the invitation to participate after their scheduled appointments and were provided with a recruitment poster if they so choose. Recruitment was further supported via snowball sampling techniques – recruited patients, medical students and physicians were invited to share recruitment information with others that they believe could help answer the research questions. |
| 12. Sample size. How many participants were in the study? | 34 |
| 13. Non-participation. How many people refused to participate or dropped out? Reasons? Setting | None. All original recruits participated. |
| 14. Setting of data collection. Where was the data collected? e.g. home, clinic, workplace | Virtually |
| 15. Presence of non-participants. Was anyone else present besides the participants and researchers? | No |
| 16. Description of sample. What are the important characteristics of the sample? e.g. demographic data, date | Medical students were in year 1 or 2 of an accredited medical school located in a large urban city in Ontario. Physicians were medical oncologists whose practice in Ontario, in both inpatient and outpatient oncology care areas. Patients were those receiving onco-logical care at a cancer center in Ontario, with varying cancer diagnoses and prognoses. |

| | |
|---|---|
| 17. Interview guide. Were questions, prompts, guides provided by the authors? Was it pilot tested? | The interview guide was developed by K.M – an experienced qualitative researcher. |
| 18. Repeat interviews. Were repeat interviews carried out? If yes, how many? | No |
| 19. Audio/visual. recording Did the research use audio or visual recording to collect the data? | Yes – interviews were conducted virtually. |
| 20. Field notes. Were field notes made during and/or after the interview or focus group? | Yes – they were conducted by D.A and reviewed by K.M during scheduled debriefs. |
| 21. Duration. What was the duration of the interviews or focus group? | 20-45 minutes |
| 22. Data saturation. Was data saturation discussed? | In staying true to our methodology, we use the term richness, not saturation. Richness in answering the research questions is achieved when researchers have moved beyond simply identifying themes and patterns to developing new insights and discoveries, which the authors believe has been done by situating the data beyond existing attribute frameworks and providing new insights from the study data in the discussion. Interpretive Description methodology also emphasizes the importance of considering data that do not fit the dominant themes or patterns; which is demonstrated by the development of additional findings that extend beyond the CanMED framework utilized. |
| 23. Transcripts returned. Were transcripts returned to participants for comment and/or correction? | No |

Domain 3: analysis and findings

| | |
|---|---|
| 24. Number of data coders. How many data coders coded the data? | 2, D.A and K.M. |
| 25. Description of the coding tree. Did authors provide a description of the coding tree? | Yes, the coding framework is included as supplemental material. |
| 26. Derivation of themes. Were themes identified in advance or derived from the data? | Both. CanMED framework was used to map data; as well as the development of two additional themes that did not fit with the CanMED framework. |

| | |
|---|---|
| 27. Software. What software, if applicable, was used to manage the data? | No, software was not used. |
| 28. Participant checking. Did participants provide feedback on the findings? | No |
| 29. Quotations presented. Were participant quotations presented to illustrate the themes / findings? Was each quotation identified? e.g. participant number | Yes. Participants were identified by their stakeholder group (medical student, physician, patient). |
| 30. Data and findings consistent. Was there consistency between the data presented and the findings? | Yes |
| 31. Clarity of major themes. Were major themes clearly presented in the findings? | Yes |
| 32. Clarity of minor themes. Is there a description of diverse cases or discussion of minor themes? | After immersive data analysis, it was determined that disaggregating the data by way of each stakeholder group (medical students, physicians, patients) was not necessary, nor would doing so enhance our understandings of the findings. Tensions, incongruence or inconsistencies between stakeholder group findings did not arise as it related to the central thematic findings |

Tong, A., Sainsbury, P., & Craig, J. (2007). Consolidated criteria for reporting qualitative research (COREQ): a 32-item checklist for interviews and focus groups. *International Journal for Quality in Health Care*, *19*(6), 349–357. https://doi.org/10.1093/intqhc/mzm042

S2: Coding framework

**Coding framework stage 1: High level examination of key attributes discussed by participants**

Empathy (n= 6): p5, p6, p17, p25, p26, p27

Compassion (n = 6): p1, p4, p7, p8, p23, p34

Caring (n =3): p15, p3; p33

Professionalism (n = 2): p2, p32

Knowledge (n = 2): p18, p21

Communication (n = 2): p19, p30

Listening (n=2): p12; p31

Patient (n = 2): p11, p24

Curiosity (n= 2): p14, p13

Trust (n = 1): p16

Kindness (n= 1): p28

Competent (n =1): p22

Skill confidence (n = 1): p29

Expert (n =1): p9

Humility (n = 1): p10

Open-minded (n = 1) p20

**Coding framework stage 2: Mapping key attributes discussed by participants to CanMED attributes**

as defined by CanMED, https://canmeds.royalcollege.ca/uploads/en/framework/CanMEDS%202015%20Framework_EN_Reduced.pdf

**CanMED Medical expert:**

Knowledge (n = 2), competent (n = 1), expert (n = 1), skill confidence (n = 1)

- Physicians (n = 2), medical students (n =2), patients (n= 1)

**CanMED Professional:**

Professionalism (n = 2), trust (n = 1)

- Physicians (n = 2), medical students (n = 1)

**CanMED Communicator:**

Communication (n = 2), listening (n = 1), patient (n = 2), and humility (n = 1)

- Physicians (n = 1), medical students (n = 1), and patients (n= 4)

**CanMED Collaborator**: 0

**CanMED Leader:** 0

**CanMED Health advocate**: 0

**CanMED Scholar**: 0

**“Others” outside of CanMED**

Compassion (n = 6), empathy (n = 6), caring (n = 3), kindness (n = 1); Open-minded (n=1) Curiosity (n= 2):

- Physicians (n = 6), medical students (n = 7), and patients (n= 6)

**Coding framework stage 3: Situating “Others”**

Caring: Compassion (n = 6), empathy (n = 6), caring (n = 3), kindness (n = 1)

- Physicians (n=4), medical students (n=6), patients (n=6)

Open-minded (n=1) and Curiosity (n= 2):

- Physicians (n=2), medical students (n=1)
